# Dysregulation of Autophagy in Murine Fibroblasts Resistant to HSV-1 Infection

**DOI:** 10.1371/journal.pone.0042636

**Published:** 2012-08-10

**Authors:** Valerie Le Sage, Bruce W. Banfield

**Affiliations:** Department of Biomedical and Molecular Sciences, Queen's University, Kingston, Ontario, Canada; Dartmouth Medical School, United States of America

## Abstract

The mouse L cell mutant, gro29, was selected for its ability to survive infection by herpes simplex virus type 1 (HSV-1). gro29 cells are fully susceptible to HSV-1 infection, however, they produce 2000-fold less infectious virus than parental L cells despite their capacity to synthesize late viral gene products and assemble virions. Because productive HSV-1 infection is presumed to result in the death of the host cell, we questioned how gro29 cells might survive infection. Using time-lapse video microscopy, we demonstrated that a fraction of infected gro29 cells survived infection and divided. Electron microscopy of infected gro29 cells, revealed large membranous vesicles that contained virions as well as cytoplasmic constituents. These structures were reminiscent of autophagosomes. Autophagy is an ancient cellular process that, under nutrient deprivation conditions, results in the degradation and catabolism of cytoplasmic components and organelles. We hypothesized that enhanced autophagy, and resultant degradation of virions, might explain the ability of gro29 to survive HSV-1 infection. Here we demonstrate that gro29 cells have enhanced basal autophagy as compared to parental L cells. Moreover, treatment of gro29 cells with 3-methyladenine, an inhibitor of autophagy, failed to prevent the formation of autophagosome-like organelles in gro29 cells indicating that autophagy was dysregulated in these cells. Additionally, we observed robust co-localization of the virion structural component, VP26, with the autophagosomal marker, GFP-LC3, in infected gro29 cells that was not seen in infected parental L cells. Collectively, these data support a model whereby gro29 cells prevent the release of infectious virus by directing intracellular virions to an autophagosome-like compartment. Importantly, induction of autophagy in parental L cells did not prevent HSV-1 production, indicating that the relationship between autophagy, virus replication, and survival of HSV-1 infection by gro29 cells is complex.

## Introduction

Alphaherpesviruses include the common human pathogens, herpes simplex virus types 1 and 2 (HSV) and varicella zoster virus (VZV), as well as many other viruses of wild and domestic animals [1]. All herpes virions share a common structure; an icosahedral nucleocapsid, containing a linear double-stranded DNA genome, surrounded by a lipid envelope embedded with a dozen or more glycoproteins [2]. Between the nucleocapsid and the envelope lies a complex proteinaceous compartment called the tegument. During infection, entry of the virion nucleocapsid and associated tegument components occurs after fusion of the virion envelope with, depending on the cell type, either the plasma membrane or an endosomal membrane. The nucleocapsid is transported along microtubules from the cell periphery towards the nucleus where it docks at a nuclear pore and the genome is injected into the nucleoplasm. Viral gene expression takes place in a temporally ordered cascade with immediate early gene products synthesized first, followed by the early and late gene products [2], [3].

The initial stages of herpesvirus assembly take place in the nucleus where newly replicated virus genomes are packaged into preformed capsids. DNA-containing capsids gain access to the cytoplasm by first acquiring a primary envelope at the inner nuclear membrane by budding into the perinuclear space. Perinuclear virions are subsequently de-enveloped through fusion of the virion envelope with the outer nuclear membrane thereby releasing the capsid into the cytoplasm. The tegument is formed through the recruitment of tegument proteins to capsid components, interactions between tegument proteins and interactions between tegument proteins and the cytoplasmic tails of membrane glycoproteins destined for the envelope of mature virions [4]. The virion acquires its final envelope through budding of capsid-tegument complexes into membranes derived from the trans-Golgi network (TGN) or possibly late endosomes (LE) [5], [6], [7]. TGN/LE derived vesicles containing infectious enveloped virus then traffic to, and fuse with, the plasma membrane of the cell, releasing virus into the extracellular environment.

While our understanding of alphaherpesvirus structure, assembly and egress has advanced considerably over the past two decades, many fundamental aspects of virus-cell interactions remain to be elucidated and this is particularly true for the contributions of cellular components to productive virus infection. As a strategy to identify cellular molecules required for the production of infectious HSV-1, Tufaro and colleagues performed a phenotypic screen searching for mutant murine L cells that could survive exposure to HSV-1 [8]. Two general classes of mutants were identified in this screen; those defective in virus entry and those that had defects in the release of infectious virus [9], [10]. The characterization of gro2C cells and its derivative, sog9, which displayed defects in the entry of HSV-1 into cells, proved to be particularly informative in establishing the role of glycosaminoglycans in the attachment of HSV-1, as well as numerous other viruses, to the cell surface [10], [11], [12], [13], [14], [15], [16], [17], [18], [19], [20], [21], [22], [23], [24], [25]. By contrast, the analysis of cellular mutants with defects in HSV-1 egress, proved to be more problematic.

gro29 cells were fully susceptible to infection by HSV-1 as well as the swine pathogen pseudorabiesvirus (PRV) [8], [26]. Moreover, both HSV-1 and PRV infected gro29 cells expressed late viral gene products efficiently, however, a striking block to viral glycoprotein transport and secretion was observed [9], [26], [27]. HSV-1 nucleocapsids assembled and were transported into the cytoplasm of gro29 cells, where these non-infectious enveloped virions accumulated inside cytoplasmic vesicles, akin to the irregular cytoplasmic vesicles that accumulate in HSV-1 infected cells treated with the ionophore monensin [28]. This defect resulted in a 2000-fold reduction in the amount of infectious HSV-1 released from gro29 cells [9]. Because HSV-1 infection of cultured cells is highly cytotoxic, the ability of gro29 cells to become infected and assemble non-infectious virions is at odds with their ability to survive exposure to HSV-1. The present study was initiated to investigate this paradox.

The process of autophagy functions to maintain cellular homeostasis by clearing damaged cellular organelles and protein aggregates from the cytoplasm [29]. Autophagy is upregulated in response to environmental stresses such as nutrient deprivation to provide a mechanism for cell survival through the catabolism of cytoplasmic constituents. Activation of elongation initiation factor 2 alpha (eIF2α kinases, such as the nutrient sensing kinase general control nondepressible 2 (GCN2) that senses amino acid deprivation, leads to phosphorylation of eIF2α [30]. This results in inhibition of translation initiation, which is a key early step in induction of autophagy [31]. Autophagosomes, which are double-membrane vesicles that envelop cytoplasmic components, fuse with lysosomes to form autolysosomes wherein the degradation of the cytoplasmic material occurs. Over the past decade, a role for autophagy in the degradation of invading intracellular pathogens has been revealed and has been termed xenophagy [32], [33], [34]. Accordingly, many successful intracellular pathogens, including HSV-1, have evolved mechanisms to evade and antagonize xenophagy to promote their survival [31], [35], [36], [37], [38], [39], [40], [41], [42]. In the case of HSV-1, the viral protein ICP34.5 prevents xenophagy by two distinct mechanisms. Firstly, ICP34.5 binds to protein phosphatase 1α and directs the dephosphorylation of eIF2α to overcome host protein shutoff [43], [44], [45]. Secondly, ICP34.5 binds directly to Beclin-1, a cellular protein required for the stimulation of autophagy and antagonizes Beclin 1-mediated autophagy [31], [35], [36], [37], [38], [39], [40], [41], [42].

Our findings indicate that infected gro29 cells are capable of cell division and that enhanced steady state levels of autophagy are observed in uninfected gro29 cells. Furthermore, HSV-1 nucleocapsids co-localize with autophagosome-like compartments in infected gro29 cells, suggesting that virions are subverted within this compartment thereby preventing the release of infectious virus from these cells.

## Results

### gro29 cells survive HSV-1 infection

gro29 cells were isolated from a population of mouse L fibroblasts that had been chemically mutagenized with ethyl methanesulfonate and challenged with a lethal inoculum of HSV-1 [8]. Since HSV-1 infection of gro29 cells progresses to late stages of virion assembly it suggested that the initial isolation of gro29 cells was possible because they either escaped the infection challenge, or that they were able to survive the cytotoxicity associated with the late stages of HSV-1 infection. To investigate this further, we recapitulated the selection of gro29 cells. To do this, we first constructed gro29 and parental L cells that stably express EGFP (gfp) and mCherry (mCh), respectively, so that the identity of individual cells in a mixed population of gro29 and L cells could be unambiguously determined. A total of one million cells were seeded at ratios of 50∶50, 90∶10 and 99∶1 (L/mCh∶gro29/gfp cells). Cells were infected at a multiplicity of infection (MOI) of 10, and after 72 hours the number and identity of surviving cells were tabulated. The average of three independent experiments performed in triplicate is shown in Table 1. L/mCh cells were unable to survive the HSV-1 infection under any condition tested. By contrast, a population of gro29/gfp cells survived infection, which was confirmed by their exclusion of trypan blue. Surviving gro29/gfp cells were generally found as single cells in isolation from each other. Seeding 500,000, 100,000 and 10,000 gro29/gfp cells in the presence of increasing numbers of L/mCh cells resulted in the survival of 90, 5 and 4 gro29/gfp cells, respectively, indicating a very low survival rate of gro29/gfp cells under conditions where productively infected L/mCh cells were also present. Strikingly, in the absence of L/mCh cells the numbers of surviving gro29/gfp cells at 72 hours was comparable to the numbers of cells plated at the beginning of the experiment and represented 10%–14% of the total number of cells seen in the mock infected samples. Taken together, these results suggest that gro29/gfp cells are capable of surviving HSV-1 infection and that in the absence of L/mCh cells, which provide a sustained source of infectious virus, substantially greater numbers of gro29/gfp cells survive.

**Table 1:** Recapitulation of gro29 selection.

Multiplicity of infection (MOI)	Number of cells plated		Number of viable cells (SD)	
	L/mCh cell	gro29/gfp cell	L/mCh cell	gro29/gfp cell
				
mock	0	5×10^5^	-	3.89×10^6^ (±6.2×10^5^)
mock	0	1×10^5^	-	1.22×10^6^ (±2.25×10^5^)
mock	0	1×10^4^	-	7.68×10^4^ (±1.68×10^4^)
10	5×10^5^	5×10^5^	0	90 (±43)
10	9×10^5^	1×10^5^	0	5 (±3)
10	9.9×10^5^	1×10^4^	0	4 (±3)
10	5×10^5^	0	0	-
10	9×10^5^	0	0	-
10	9.9×10^5^	0	0	-
10	0	5×10^5^	-	3.7×10^5^ (±1.7×10^5^)
10	0	1×10^5^	-	1.28×10^5^ (±4.22×10^4^)
10	0	1×10^4^	-	1.06×10^4^ (±5.05×10^3^)

**Figure 1:**
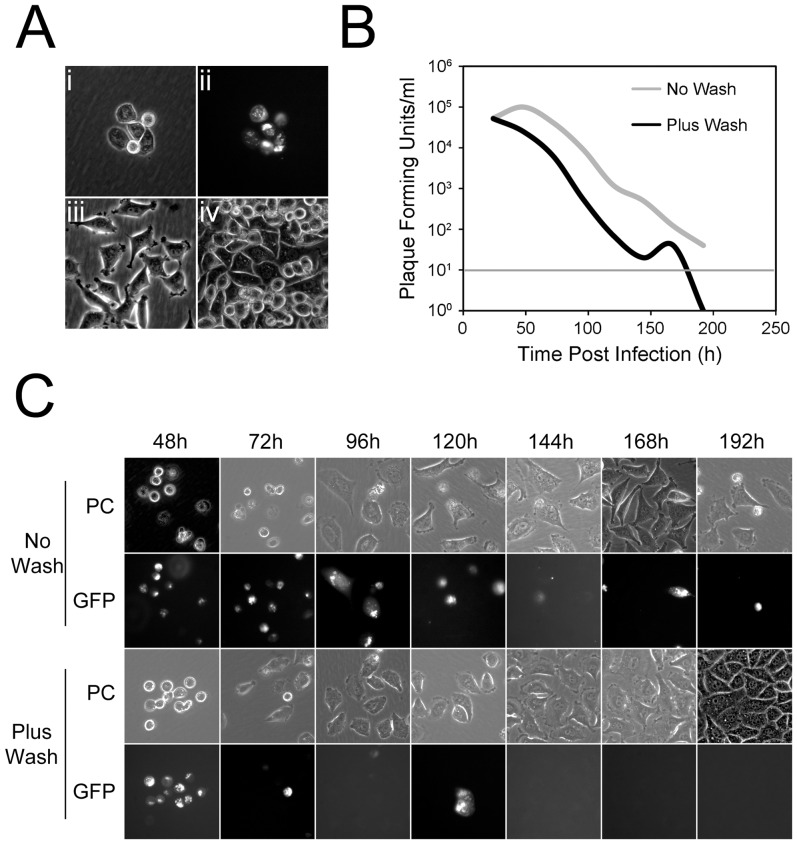
Time course of gro29 cell survival and infection. (A) gro29 cells were infected with HSV-1 Us2-GFP at an MOI of 30 at 24 h post infection. Representative phase contrast (i) with corresponding GFP fluorescence (ii) images are shown. Phase contrast images of mock infected gro29 cells at 24 h (iii) and 192 h (iv). (B) The amount of virus produced from HSV-1 infected gro29 cells in the absence (grey line) or presence (black line) of a low pH wash every 24 h. At the indicated times post infection total infectious virus was quantified by titration on Vero cells and calculated as the number of plaque forming units (PFU) per mL. The sensitivity of the assay was 10^1^ plaque forming units and is indicated by the thin horizontal grey line. (C) Time course of infected gro29 cells in the absence (No Wash) or presence (Plus Wash) of a low pH citrate buffer wash every 24 h. Representative phase contrast (PC) with corresponding GFP fluorescence images are shown.

To provide a comprehensive analysis of gro29 cell infection over time, we performed an extended time course in which gro29 cell survival, expression of virus antigen and virus production were monitored. To ensure that all gro29 cells were initially infected, an MOI of 30 was used. To monitor the expression of virus antigen, cells were infected with a recombinant HSV-1 strain that expresses a Us2-EGFP fusion protein. At the indicated times post infection, cell survival was evaluated by phase contrast microscopy (Fig. 1A,C), HSV-1 infection was visualized by EGFP fluorescence (Fig. 1A,C) and the production of virus was quantified by titration of total virus present in the cultures (Fig. 1B). Microscopic examination of cells at 24 h post-infection confirmed that all of the gro29 cells were infected at the beginning of the experiment (Fig. 1Aii). At 48 h and 72 h post infection, the majority of gro29 cells demonstrated a rounded up morphology and contained viral antigen (Fig. 1C). The proportion of EGFP positive gro29 cells decreased progressively over time until 144 h post infection when isolated infected gro29 cells persisted in the population, however, the majority of cells in the culture demonstrated a flat morphology and were negative for virus antigen (Fig. 1C). Consistent with these findings, virus production decreased precipitously overtime with only trace amounts of infectious virus detected at 192 h post infection (Fig. 1B, no wash). These findings suggested that low levels of virus were able to persist in the cultures, presumably due to a low frequency of HSV-1 re-infection of cells. To further explore this idea, a similar experiment was performed with the exception that secondary spread of virus in the cultures was prevented by eliminating extracellular virus every 24 h by replacement of culture medium and brief exposure of cells to low pH citrate buffer. Under these conditions the numbers of cells, expressing virus antigen was reduced dramatically by 72 h post-infection and was undetectable after 144 h. Between 72 h and 192 h the gro29 cells regained a flat morphology similar to that seen in uninfected cell cultures (Fig. 1Aiii, iv). The amount of virus produced by these cultures decreased steadily over time and was below the level of detection by 168 h post infection (Fig. 1B). Collectively these data indicate that gro29 cells are able to survive infection by HSV-1 and that blocking HSV-1 re-infection of cells facilitates the clearance of virus from the cultures.

**Figure 2:**
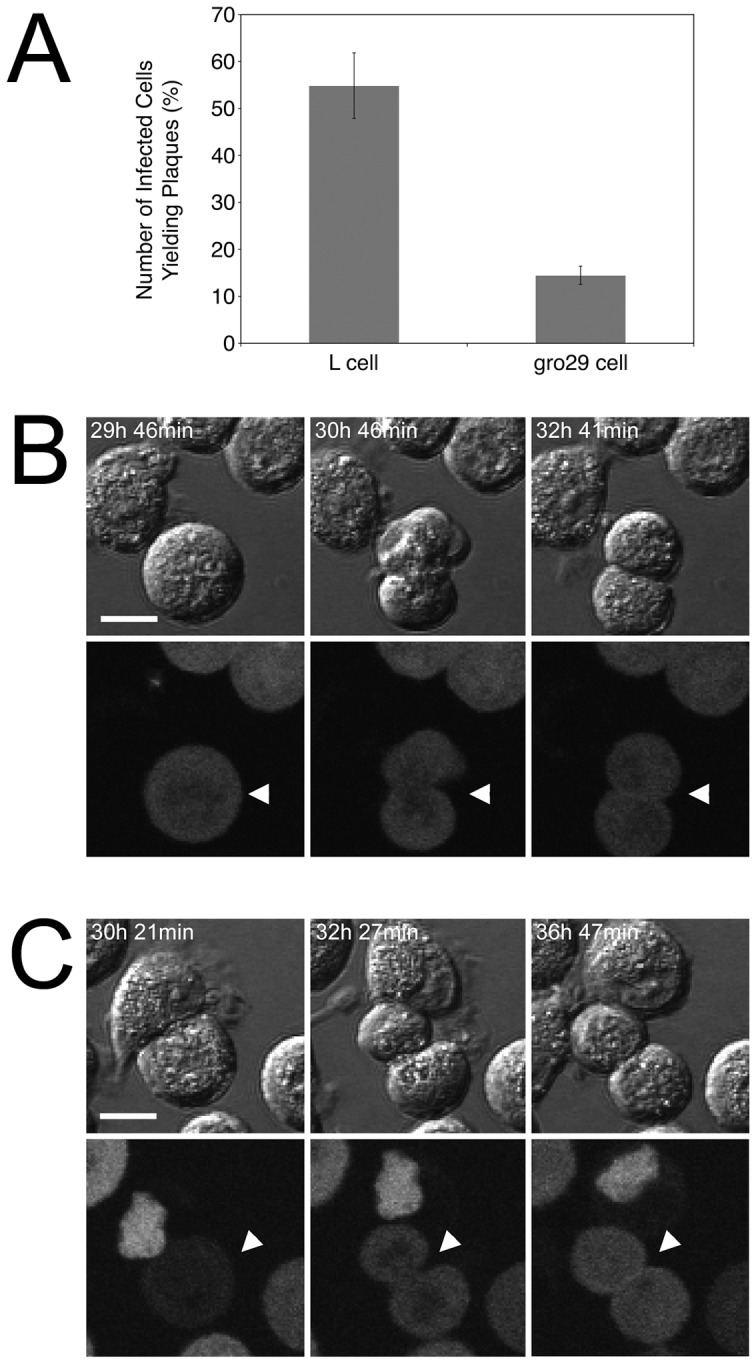
gro29 cells are able to survive HSV-1 infection. (A) The percentage of L and gro29 cells able to transmit virus infection was determined by infectious centre assays as described in Materials and Methods. The results are expressed as an average of infected cells capable of plaque formation ± standard deviation (S.D.) of three independent experiment performed in triplicate. (B, C) gro29 cells were infected with HSV-1 Us2-GFP and imaged by time-lapse microscopy. Infected gro29 cells (arrowheads) were observed to divide, representative images of two different events are shown at the indicated times post infection. DIC images are shown in top panels with corresponding GFP images below. Scale bar represents 10 µm.

gro29 cells harbor a defect in HSV-1 virion egress that results in low yields of infectious virus [8], [9]. Given that HSV-1 infection and virus production result in the destruction of the host cell, we asked whether the low levels of virus produced by gro29 cells was associated with all infected cells expelling a small amount of virus, or a small population of infected gro29 cells producing a larger amount of virus. To distinguish between these possibilities infectious centre assays were performed for both L and gro29 cells (Fig. 2A). Infected L and gro29 cells were harvested at 3 h post infection, diluted and inoculated directly onto Vero cell monolayers. Roughly, 54% of infected L cells were capable of forming plaques on Vero cells as compared to 14% of infected gro29 cells. These findings indicate that, unlike L cells, the majority of infected gro29 cells do not produce detectable levels of infectious virus. However, given the numbers of gro29 cells that survive infection and how little infectious virus is produced by the population, it was remarkable that as compared to L cells nearly 25% of infected gro29 cells were capable of completing the virus replicative cycle.

To determine if infected gro29 cells were capable of dividing, gro29 cells infected with recombinant HSV-1 strains that express Us2-EGFP (Fig. 2B,C), or gB-EGFP (data not shown) fusion proteins, were monitored by time-lapse video microscopy. Whereas L cells infected with these strains were observed to round up and die (data not shown), infected gro29 cells were observed to divide (Fig. 2B,C). These remarkable findings indicate that gro29 cells expressing late HSV-1 gene products retain the capacity to divide.

### Autophagy is upregulated in gro29 cells

**Figure 3:**
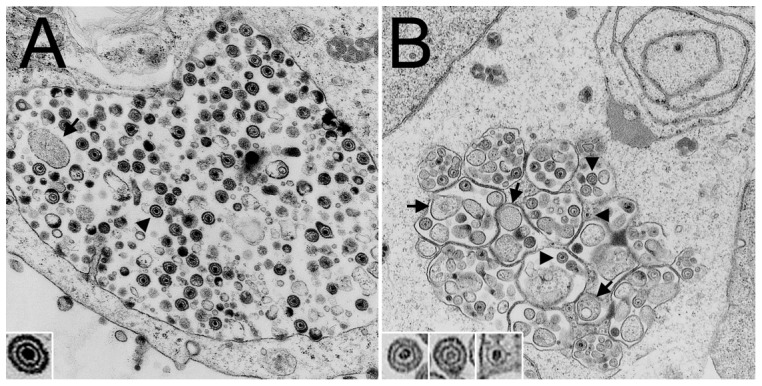
gro29 cells accumulate virions in vesicles reminiscent of autophagosomes. At 18 h post infection, HSV-1 infected L (A) and gro29 (B) cells were harvested for electron microscopy. Representative electron micrographs are shown. Arrowheads indicate virions, while arrows identify cytoplasmic material within the vesicles. Inset in (A) shows a mature HSV-1 virion. Insets in (B) show a mature virion, an enveloped empty capsid and a non-enveloped nucleocapsid containing DNA (left to right).

To provide clues to the block to virus production seen in gro29 cells, transmission electron microscopy (TEM) was performed. A previous TEM analysis showed that the ultrastructure of infected gro29 cells differed considerably from that of infected L cells [9]. Consistent with previous findings, re-examination of infected L cells by TEM revealed large vesicles containing enveloped virions bearing electron dense cores indistinguishable from extracellular enveloped infectious virions (Fig. 3A). Similarly, micrographs of infected gro29 cells showed many smaller vesicles containing fewer enveloped viral particles (Fig. 3B). In addition to intact virions, enveloped empty capsids as well as non-enveloped DNA-containing capsids associated with these structures (Fig. 3B, insets). Moreover, the vesicles within infected gro29 cells appeared similar to autophagosomes insofar as they contained a great deal of cytoplasmic material (Fig. 3B, arrows). We next investigated the status of autophagy in L and gro29 cells.

**Figure 4:**
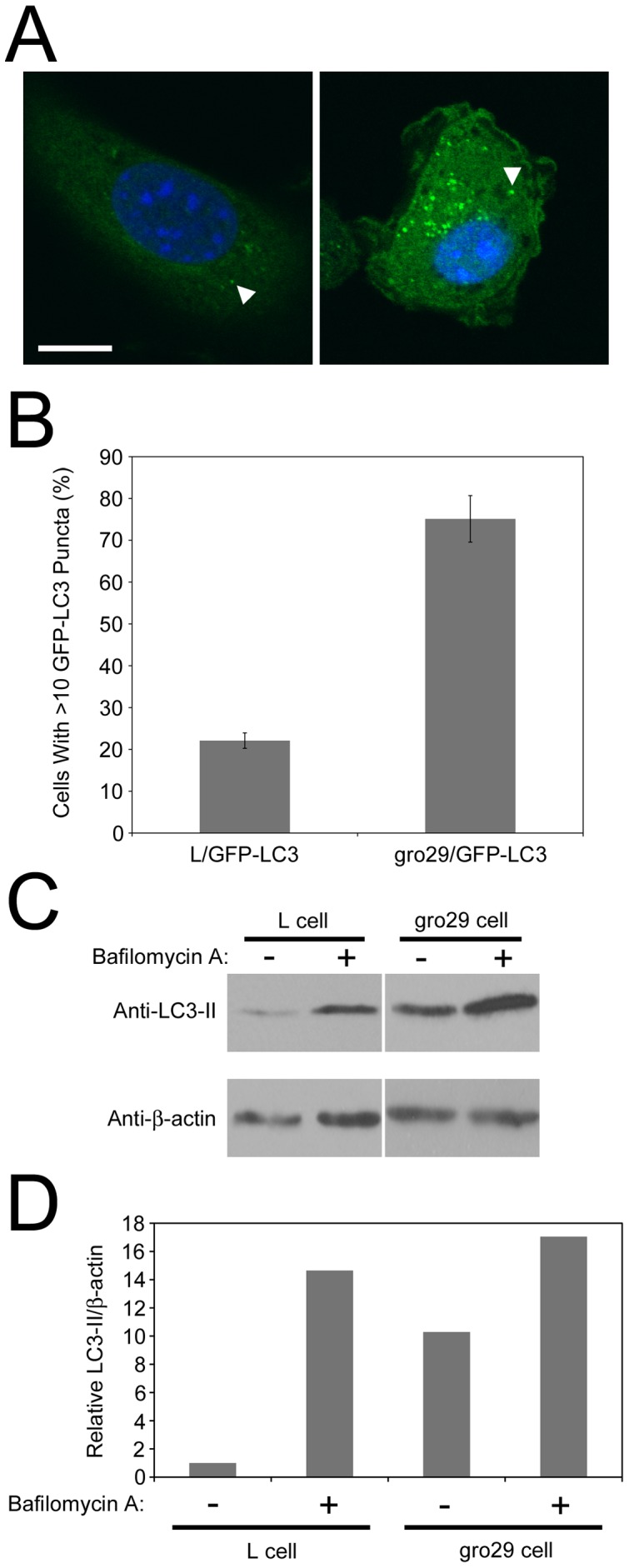
gro29 cells have increased autophagosome formation under non-autophagy inducing conditions. (A) Representative confocal images of L/GFP-LC3 (left panel) and gro29/GFP-LC3 (right panel) under nutrient rich conditions. Nuclei were stained with Hoechst 33342. The arrowhead indicates an autophagosome. Scale bar is 10 µm. (B) The percentage of cells having induced autophagy was determined as the percentage of L/GFP-LC3 and gro29/GFP-LC3 cells with greater than 10 GFP-LC3 puncta per cell. The data shown represent an average ± S.D. from three independent experiments. (C) Western blotting analysis of LC3-II and β-actin. L and gro29 cells were incubated in the absence or presence of 50 nM bafilomycin A and cells lysates were prepared after 4 h. Equal volumes of cell extract were analyzed by Western blotting with the indicated antibodies. Data are representative of three independent experiments. Panels shown for each antibody were from the same blot and same exposure, they have been separated in the figure to remove unrelated lanes. (D) The corresponding quantitation of the expression levels of LC3-II and β-actin in L and gro29 cells of panel C by densitometry using NIH ImageJ v1.44. Ratios of LC3-II to β-actin were determined and normalized to L cells in the absence of bafilomycin A, which was arbitrarily set to a value of 1.0.

Autophagosome formation requires microtubule-associated protein 1 light chain 3 (LC3), which is commonly used as a marker of autophagy [46]. In resting cells, LC3 is found in the cytoplasm in a soluble form called LC3-I. Upon induction of autophagy, LC3-I is conjugated to the lipid phosphatidylethanolamine to form LC3-II, which targets it to autophagosomal membranes. This membrane-bound form of LC3 participates in the formation of autophagosomes and remains tethered to mature autolysosomes, where intra-luminal LC3-II is degraded along with the other luminal contents [47], [48]. Consequently, LC3-II is undergoing continuous turnover [49]. To examine the autophagic state of L and gro29 cells we constructed L/GFP-LC3 and gro29/GFP-LC3 cell lines that stably express GFP-LC3 [50] and incubated these cells under nutrient rich conditions that do not induce autophagy. Cells possessing more than 10 GFP-LC3 puncta were scored as having enhanced autophagy. In L/GFP-LC3 cells, GFP-LC3 localization was mostly diffuse throughout the cytoplasm with approximately 78% of L/GFP-LC3 cells having fewer than 10 GFP-LC3 puncta (Fig. 4A,B). By contrast, 75% of gro29/GFP-LC3 cells growing under nutrient rich conditions displayed a punctate GFP-LC3 distribution that was markedly increased as compared to the L/GFP-LC3 cell line, suggesting enhanced basal levels of autophagy in gro29/GFP-LC3 cells (Fig. 4A,B).

It is possible that the accumulation of GFP-LC3 puncta in gro29/GFP-LC3 cells was due to a block in autophagosome maturation and turnover [51], [52]. The cellular level of LC3-II undergoes continuous turnover as autophagosomal membrane-bound LC3-II is degraded by lysosomal hydrolases following lysosomal fusion and autophagosomal maturation into autolysosomes [49]. Degradation of LC3-II can be inhibited by bafilomycin A, which prevents the transition of autophagosomes into autolysosomes by inhibition of the vacuolar type H(+)-ATPase [53]. If gro29 cells experienced a block in autophagosomal maturation we would expect that bafilomycin A treatment would have no effect on the levels of LC3-II. However, if gro29 cells have enhanced basal autophagy then the levels of LC3-II should be higher in gro29 cells than L cells in the absence of bafilomycin A and should increase upon exposure to this drug. L and gro29 cells were incubated under nutrient rich conditions in the presence or absence of bafilomycin A for 4 h. Cell lysates were collected and the levels of LC3-II monitored by immunoblotting (Fig. 4C). LC3-II levels were higher in untreated gro29 cells than in untreated L cells and treatment with bafilomycin A caused a significant accumulation of LC3-II in both L and gro29 cells (Fig. 4C,D). These results are consistent with the idea that autophagy in gro29 cells is not impaired, but that these cells display enhanced basal autophagy as compared to parental L cells.

### Autophagy is dysregulated in gro29 cells

**Figure 5:**
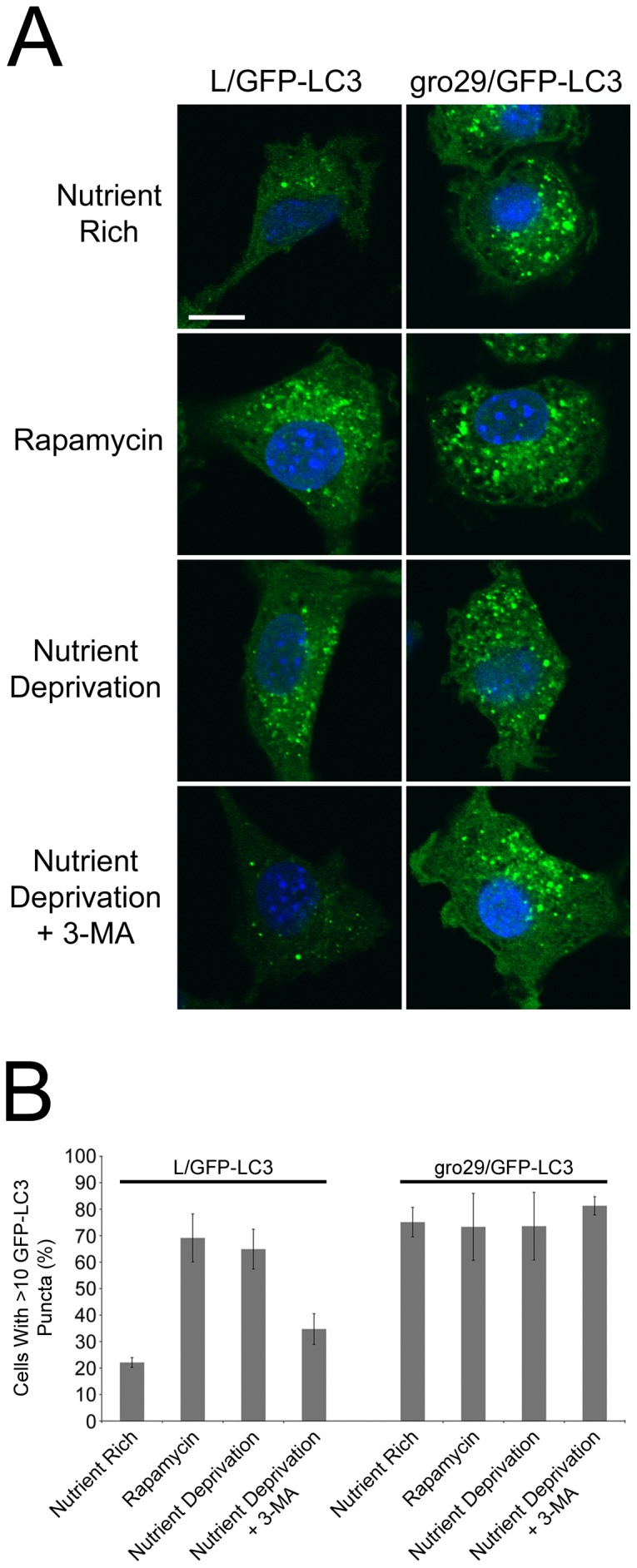
Autophagy is dysregulated in gro29 cells. (A) L/GFP-LC3 and gro29/GFP-LC3 cells were incubated for 4 h under nutrient rich conditions (DMEM/10%FBS) with or without 500 nM rapamycin, or under nutrient deprivation conditions (DMEM without FBS) with or without 5 mM 3-MA. Nuclei were stained with Hoechst 33342. Scale bar is 10 µm. (B) The percentage of cells having induced autophagy was determined as the percentage of L/GFP-LC3 and gro29/GFP-LC3 cells with greater than 10 GFP-LC3 puncta per cell. Random fields of cells (n>100 cells/condition) were quantified and confocal images are representative from three independent experiments.

Autophagy is dynamic and controlled by a number of key regulators including the mammalian target of rapamycin (mTOR). The serine/threonine kinase mTOR suppresses autophagy when nutrients are available, while inhibition of mTOR by the small molecule rapamycin results in induction of autophagy [54]. Autophagy can also be repressed using 3-methyladenine (3-MA), which blocks formation of autophagosomes via the inhibition of type III phosphatidylinositol 3-kinases (PI-3K) [55]. To investigate the regulation of autophagy in gro29 cells, we characterized the responses of L and gro29 cells to rapamycin and 3-MA. L/GFP-LC3 and gro29/GFP-LC3 cells were incubated for 4 h under nutrient rich conditions with or without rapamycin, or under nutrient deprivation conditions with or without 3-MA. The distribution of GFP-LC3 was monitored by confocal microscopy. In comparison to growth under nutrient rich conditions, the numbers of GFP-LC3 puncta and the proportion of autophagic L/GFP-LC3 cells were dramatically increased by rapamycin treatment and upon exposure to nutrient deprivation (Fig. 5A,B). Conversely, the inhibitor 3-MA was able to block the activation of autophagy in L/GFP-LC3 cells by serum deprivation as expected (Fig. 5A,B). The proportion of gro29/GFP-LC3 cells with greater than 10 GFP-LC3 puncta remained unchanged under conditions that either stimulated or repressed autophagosome formation in L/GFP-LC3 cells (Fig. 5A,B). As an alternative approach to inhibit autophagy in gro29 cells we utilized siRNAs directed against Atg7 and Atg12, two proteins that are essential for autophagosome formation and have been successfully targeted by other researchers to inhibit autophagy [56], [57]. This approach was not viable in either L or gro29 cells and resulted in marked cytotoxicity. Nonetheless, these data indicate that gro29 cells are refractory to pharmacological agents that either stimulate or repress autophagosome formation.

### Virion proteins co-localize with autophagosomes in infected gro29 cells

**Figure 6:**
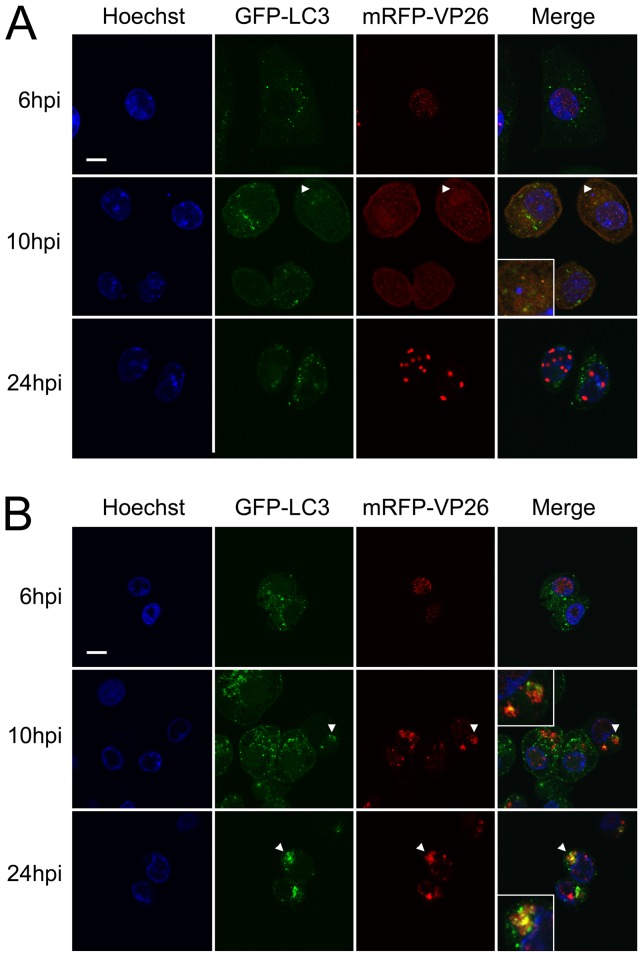
Co-localization of autophagosomes and viral capsids in HSV-1 infected gro29 cells. L/GFP-LC3 (A) and gro29/GFP-LC3 (B) cells were infected with HSV-1 mRFP-VP26 at an MOI of 10. At 6, 10 and 24 h post infection, the infected cells were fixed and the nuclei were stained with Hoechst. Arrowheads indicate areas in which the mRFP and the EGFP signals co-localize. Insets in the merged panels are magnified regions corresponding to the arrowheads. Confocal images are representative of three independent experiments.

The data so far suggested that virions in gro29 cells accumulated in what appeared to be autophagosome-like compartments (Fig. 3B). This finding, coupled with the observation that gro29 cells have enhanced basal autophagy (Fig. 4), lead us to investigate the localization of the autophagosomal marker LC3 and virion structural components. L/GFP-LC3 and gro29/GFP-LC3 cells were infected with HSV-1 mRFP-VP26 [58] at an MOI of 10 and visualized by confocal microscopy. Consistent with HSV-1 inducing autophagy in infected cells [59], early during the time course of L cell infection with HSV-1 mRFP-VP26, the number of GFP-LC3 puncta increased (Fig. 6A). At 10 h post infection, L/GFP-LC3 cells contained few cytoplasmic GFP-LC3 puncta with 5.4% of infected cells having co-localization with mRFP-VP26 (Fig. 6A, arrowheads), however by 24 h post infection co-localization of the two markers was apparent in 1.7% of infected L/GFP-LC3 cells. In gro29/GFP-LC3 cells, mRFP-VP26 accumulated in large cytoplasmic vesicles as the infection progressed (Fig. 6B). Progression of the HSV-1 mRFP-VP26 infection in gro29/GFP-LC3 cells caused a redistribution of GFP-LC3 from isolated GFP-LC3 puncta to larger vesicular structures that covered a greater proportion of the cytoplasm (Fig. 6B). At 10 h and 24 h post infection, 15 and 23.5% of infected gro29/GFP-LC3 cells possessed many of these large cytoplasmic GFP-LC3 positive structures that co-localized with mRFP-VP26 (Fig. 6B, arrows). These results suggest that virion structural components accumulate in autophagosome-like compartments in gro29/GFP-LC3 cells.

### The phosphorylation status of eIF2α is unaffected in gro29 cells

**Figure 7:**
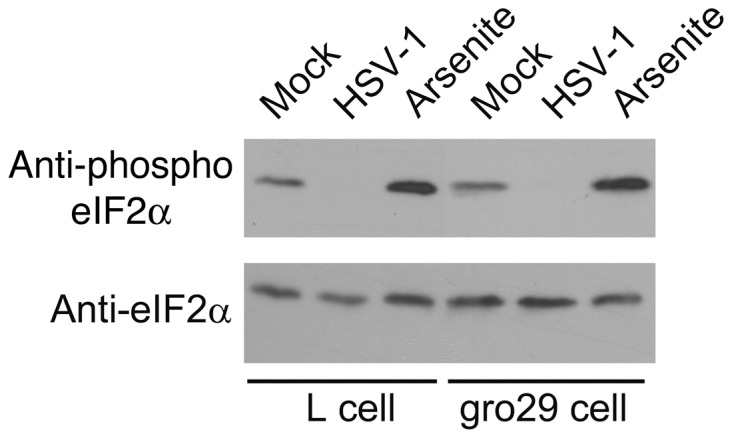
Phosphorylation status of eIF2α in L and gro29 cells. L and gro29 cells, cells were mock-infected for 6 h, mock infected for 6 h then treated with 0.5 mM sodium arsenite for 30 min to stimulate eIF2α phosphorylation, or infected with HSV-1 at an MOI of 10 for 6 h. Equal volumes of whole cell lysate were electrophoresed through 10% polyacrylamide gels and transferred to PVDF membranes. Membranes were probed with antisera indicated on the left.

The HSV-1 ICP34.5 protein can inhibit autophagy by promoting the dephosphorylation of eIF2α [31], [43], [44], [45]. A possible explanation for the accumulation of autophagosome-like compartments in infected gro29 cells is the failure of ICP34.5 to reverse eIF2α phosphorylation. To examine the phosphorylation status of eIF2α in L and gro29 cells, cells were mock-infected for 6 h, mock infected for 6 h then treated with 0.5 mM sodium arsenite for 30 min to stimulate eIF2α phosphorylation, or infected with HSV-1 at an MOI of 10 for 6 h. Whole cell lysates were prepared and analyzed by Western blotting using antisera against phospho-eIF2α or total eIF2α (Fig. 7). The levels of phospho-eIF2α in uninfected L and gro29 cells were similar. The levels of phospho-eIF2α in HSV-1 infected L and gro29 cells were reduced compared to uninfected cells, which is consistent with the activity of ICP34.5. Moreover, treatment of both L and gro29 cells with sodium arsenite stimulated eIF2α phosphorylation. Taken together, these data indicate that the phosphorylation status of eIF2α in gro29 cells is regulated normally in infected and uninfected cells. These findings suggest that increased phosphorylation of eIF2α is not responsible for the enhanced basal autophagy observed in uninfected gro29 cells, or the accumulation of autophagosome-like compartments in infected gro29 cells.

### Induction of autophagy in L cells does not recapitulate the gro29 phenotype

**Figure 8:**
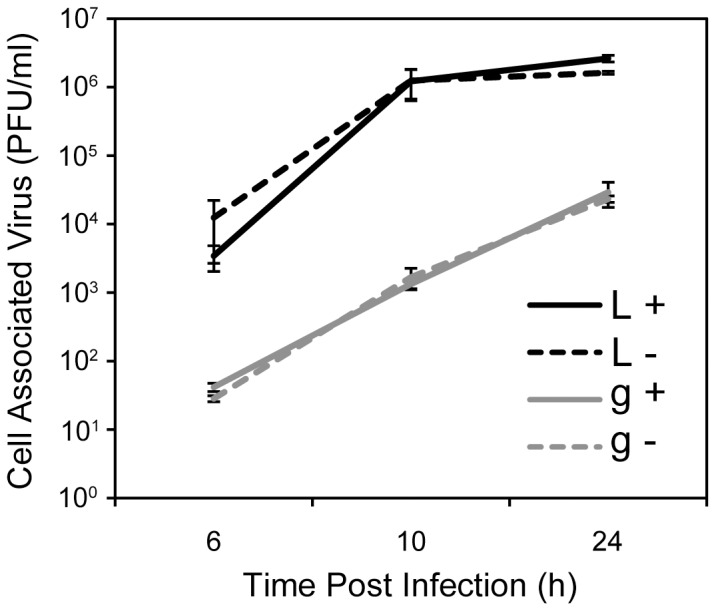
Induction of autophagy in L cells does not reduce the production of intracellular HSV-1. L (L) and gro29 (g) cells were pre-incubated for 1 h and maintained under nutrient rich (DMEM/10%FBS) (+) or nutrient deprivation (DMEM without FBS) (−) conditions and challenged with HSV-1 at an MOI of 1. At the indicated times post infection the cell associated virus was collected and titred on Vero cells. Results are from three independent experiments, with S.D. indicated.

We hypothesized that enhanced basal autophagy in gro29 cells contributed to the lack of infectious HSV-1 virions produced in these cells. To assess whether induction of autophagy in L cells could mimic the gro29 cell phenotype, L cells were pre-incubated under nutrient deprivation conditions (Fig. 5A), prior to being infected with HSV-1 at an MOI of 10. Cell-associated virus was collected at 6, 10 and 24 h post infection and titred on Vero cells. The induction of autophagy had no effect on virus yield at any time point tested (Fig. 8). Similarly, pre-treatment of L cells with rapamycin yielded comparable results (data not shown). Moreover, further stimulation of autophagy in gro29 cells did not result in reduced cell-associated viral titres (Fig. 8). These findings suggest that nutrient deprivation did not block productive virion replication upon infection of L cells and was insufficient to recapitulate the gro29 cell phenotype.

## Discussion

gro29 cells are one of several mutant cell lines isolated from a population of chemically mutagenized L cells using HSV-1 as a selective agent [8]. The goal of this approach was to isolate cells that harbored specific defects in the ability to support HSV-1 replication. The subsequent identification of pathways, processes and genes responsible for resistance to virus infection was expected to provide clues to cellular functions required for virus infection and potentially reveal new cellular targets for antiviral interventions [24], [60], [61]. Several mutant cell lines isolated from this screen proved to have defects in HSV-1 binding to the cell surface [10], [12], [13], [14], [16], [18], [21], [22], [23], [24]. Based on the nature of the selection for HSV-1 resistant cells, it was perhaps not surprising to isolate cellular mutants defective for virus entry. HSV infection is cytotoxic to L cells and productively infected cells ultimately die, so it follows that if cells did not become infected they should survive exposure to virus. The observation that gro29 cells were fully susceptible to HSV-1 infection and could assemble non-infectious virions was contrary to the idea that virus infected cells were destined to die. Two possible mechanisms were proposed to explain the ability of gro29 cells to survive selection [8]. Since infected gro29 cells produced very little virus, one possibility was that cells at the periphery of a gro29 cell micro-colony could insulate and protect gro29 cells within the colony from infection. In support of this idea, recapitulation of the gro29 cell selection demonstrated that a small proportion of gro29 cells have the ability to resist HSV-1 infection when cells capable of amplifying virus are also present suggesting that gro29 which clear and survive initial infection can become superinfected and ultimately succumb. By contrast, when infected in isolation, greater than 1000 times more gro29 cells survived challenge with HSV-1. An alternative possibility to explain the ability of gro29 cells to survive selection with HSV-1 was that infected gro29 cells were capable of clearing the virus infection and dividing. Here we demonstrate that infected gro29 cells are indeed capable of cell division and that fully infected cultures of gro29 cells eventually cease to produce infectious virus, clear virus antigen and reform a cell monolayer indistinguishable from mock-infected cells. Thus, it might be that both insulation from infection and division of infected cells contributed to the isolation of gro29 cells.

Infectious centre assays revealed that approximately 25% of HSV-1 infected gro29 cells are able to transmit virus infection to an uninfected cell. Intriguingly, a previous analysis demonstrated that 20% of infected gro29 cells had equivalent levels of cell surface HSV-1 glycoprotein D as L cells, whereas the remaining cells had considerably reduced levels [9]. It is tempting to speculate that the population of HSV-1 infected gro29 cells with “normal" levels of glycoprotein D on their cell surface are the same population that are capable of transmitting infection. Based on their analysis of PRV infection of gro29 cells, Enquist and colleagues suggested that a cellular factor was limiting in gro29 cells because infected cells produced virus early after infection and then stopped [26]. Taken together, these findings suggest that the fate of the infected gro29 cell is variable. Whether gro29 cells produce virus, or survive infection, may depend on stochastic phenomena such as the levels of a particular cellular factor, or the stage of the cell cycle at the time of infection.

Autophagy is not only important for the degradation of organelles and long-lived proteins but also for the engulfment of virus particles by autophagosomes and autolysosomes, termed xenophagy [62], [63]. We have demonstrated that gro29 cells have an enhanced basal level of autophagy, as evidenced by an accumulation of the membrane bound form of the autophagosomal marker LC3 by Western blotting and the large number of GFP-LC3 puncta seen by microscopy. Additionally, examination of LC3-II levels in the presence and absence of bafilomycin A demonstrated that LC3-II was being turned over in gro29 and therefore this apparatus is presumably available to interfere with HSV-1 virion production. Furthermore, co-localization of LC3 and viral capsids in perinuclear cytoplasmic structures suggests that virions are contained within autophagosome-like compartments in gro29 cells. There is precedence for autophagy determining the fate of herpesvirus-infected cells. Suárez and colleagues demonstrated that the outcome of gammaherpesvirus 68 infection of endothelial cells can be regulated by autophagy; induction of autophagy promoted survival of infected cells and inhibition of autophagy promoted the death of infected cells [56]. We hypothesize that gro29 cells survive infection and produce much less infectious virus than L cells because of their enhanced autophagic state. However, other defects in virion maturation observed in gro29 cells such as aberrant processing of viral envelope glycoproteins could be responsible for the lack of infectivity associated with virons recovered from gro29 cells [8], [9]. Similar to what is observed in gro29 cells, treatment of HSV-1 infected cells with the ionophore monensin results in inhibition of viral glycoprotein processing, prevention of virion egress and the accumulation of virions in cytoplasmic vesicles [28]. In contrast to the situation in infected gro29 cells, monensin treatment of HSV-1 infected cells had only a modest effect on the infectivity of cell-associated virus suggesting that the appropriate processing of glycans on viral glycoproteins is not required for virion infectivity [28]. Thus, we favor the notion that retention of HSV-1 virions within autophagosome-like compartments leads to their degradation and loss of infectivity.

In addition to defects in the propagation of HSV-1, gro29 cells display other deficiencies that may be related to their enhanced basal level of autophagy. gro29 cells are resistant to the cytotoxic lectins, ricin and modeccin [27]. Resistance to these lectins was suggested to be due to reduced numbers of lectin-binding moieties expressed by gro29 cells as a result of impaired protein secretion and glycoprotein processing. Interestingly, sensitivity of cells to ricin is inhibited by 3-MA, suggesting that autophagy is necessary for ricin toxicity [64]. It may be that the dysregulation of autophagy observed in gro29 cells contributes to their lectin resistance. gro29 cells also display defects in the presentation of specific antigens to CTL by MHC Class I [65], [66]. Zhang and colleagues suggested that this defect was due to a loss of a protease activity in gro29 cells that resulted in the failure to generate the epitopes under investigation [66]. Alternatively, autophagosomal degradation and destruction of these epitopes in gro29 cells might explain the reduced efficiency of their presentation.

It is possible that autophagy is not responsible for gro29 survival, but rather a consequence of survival. Initial studies on HSV and PRV replication in gro29 cells revealed defects in protein secretion and viral glycoprotein processing and transport. Failure of viral glycoproteins to reach the TGN and LE where virion maturation is proposed to occur may lead to the formation of aberrant virus assembly complexes that are subsequently degraded by autophagy. Alternatively, it seems plausible that the gro29 cell defect that causes inhibition of HSV-1 egress also results in elevated levels of basal autophagy. A number of mutant cells have been shown to possess enhanced basal autophagy. For example, mutations in Ras proteins cause malignant cell transformation that requires enhanced basal autophagy for tumor cell survival under conditions of stress and to maintain homeostasis during tumorgenesis [67], [68], [69], [70]. Constitutive expression of the EJ-ras oncogene in rat embryo fibroblasts has been associated with inhibition of HSV-1 multiplication, however, unlike gro29 cells, this inhibition was found to be at the level of immediate early gene transcription [71]. Mutations in ESCRT (endosomal sorting complexes required for transport) have also been associated with an increased number of autophagosomes in the absence of autophagy inducing conditions [72]. Although, the precise mechanism of ESCRT involvement with autophagy is unknown, it is clear that functional ESCRT is required for secondary envelopment of HSV-1 [73], [74], [75]. The existence of enhanced autophagy in gro29 cells prior to infection suggests that autophagy is a solution to dealing with infection in gro29 cells rather than a byproduct of the accumulation of aberrant virion assembly intermediates.

We hypothesized that if enhanced basal autophagy in gro29 cells was responsible for resistance to HSV-1 infection then inhibition of gro29 cell autophagy should render the cells more susceptible to infection and increase the production of infectious virus. Attempts to pharmacologically inhibit autophagy in gro29 cells with 3-MA failed despite the ability of 3-MA to prevent autophagy in nutrient deprived L cells. Conversely, we rationalized that induction of autophagy in L cells might inhibit the production of HSV-1. However, neither nutrient deprivation, nor treatment with rapamycin, had an impact on the ability of HSV-1 to replicate in either L or gro29 cells. It may be that HSV-1 gene products such as ICP34.5 were able to reverse the effects of nutrient starvation in L cells. Interestingly, our data suggest that ICP34.5 was able to abrogate the amount of phospho-eIF2α in infected gro29 cells, indicating that inhibition of eIF2α dephosphorylation by ICP34.5 was not a contributing factor to gro29 cell survival.

In conclusion, our data support a model whereby gro29 cells prevent the release of infectious virus by trapping intracellular virions within autophagosome-like compartments. Conditions known to induce autophagy in parental L cells did not prevent HSV-1 production, indicating that the relationship between autophagy, virus replication, and survival of HSV-1 infection by gro29 cells is complex. Elucidation of the defect in gro29 cells that leads to its enhanced basal autophagy will provide new insight into the regulation of autophagy and how it contributes to intrinsic cellular antiviral defenses.

## Materials and Methods

### Cells and viruses

The parental cell line was clone 1D of the LMtk^−^ mouse fibroblast line (L cells), a kind gift from Dr. Frank Tufaro, University of British Columbia [8]. Ethyl methanesulfonate mutagenesis and subsequent challenge with HSV-1 of the L cell line produced the mutant gro29 cell line, which was able to survive HSV-1 infection [8]. gro29 cells were provided by Dr. Frank Tufaro. The Phoenix-Amphotropic cells were created by Dr. Gary Nolan [76], Stanford University and kindly provided by Dr. Craig McCormick, Dalhousie University. The African green monkey kidney cell line (Vero) were purchased from ATCC. All of the cell lines were grown in Dulbecco's modified Eagle's medium (DMEM) supplemented with 10% fetal bovine serum (FBS) and 1% penicillin/streptomycin.

The virus used in this study, HSV-1 strain F derived from the pYEbac102 [77], was kindly provided by Dr. Yasushi Kawaguchi, University of Tokyo. All viruses were propagated and titred on Vero cells.

### Virus construction

The dual-fluorescence virus, HSV1-GS2843, encodes mRFP1 fused to the N-terminus of the VP26 capsid protein and GFP fused to the C-terminus of the gD envelope glycoprotein was kindly provided by Dr. Greg Smith, Northwestern University [58]. The HSV-1 encoding GFP fused the N-terminus of the tegument protein Us2 was constructed by the two-step Red-mediated mutagenesis procedure [78] using the HSV-1 strain F bacterial artificial chromosome (pYEbac102) in *Escherichia coli* GS1783 using the primers 5′-CAAGTGCCCCAAATCGGACACGGGCCTGTAATATACCAACATGGTGAGCAAGGG CGAG-3′ and 5′-TCTGGTCAAGGAGGGTCATTACGTTGACGACAACAACGCCCTTGT ACAGCTCGTCCATG-3′.

### Isolation of stable cell lines

To isolate L/mCh and gro29/gfp, L and gro29 cells were transfected using FuGene 6 with the plasmids pJR70 [79] and pEGFP-C1 (Clontech, Mountain View, CA), respectively. After growth in medium containing 1 mg/mL G418 (Sigma, St. Louis, MO), drug-resistant colonies were screened by immunofluorescence, isolated and maintained in medium supplemented with 1 mg/mL G418. The L/GFP-LC3 and gro29/GFP-LC3 stable cells were isolated using an amphotropic Phoenix-MMULV system [76] and the plasmid pBMN-GFP-LC3B derived in part from pEGFP-LC3 (plasmid 11546, Addgene, Cambridge, MA, kindly provided by Dr. Karla Kirkegaard, Stanford University [38]), provided by Dr. Craig McCormick, Dalhousie University. After transduction, the cells were selected with 2 µg/mL puromycin (Invitrogen, Burlington, ON) and the expression of GFP-LC3 was confirmed by fluorescence microscopy. Cells were maintained in medium supplemented with 2 µg/mL puromycin.

### Transmission electron microscopy

Cell were grown on Millicell HS inserts (Millipore, Billerica, MA) for 24 h prior to HSV-1 (MOI = 5). At 18 hpi, cells were rinsed with PBS and monolayers were fixed in 2.5% glutaraldehyde in 0.1 M sodium cacodylate (pH 7.3) for 1 h on ice. Cells were then rinsed and postfixed in 1% OsO_4_ for 1 h. These samples were rinsed, dehydrated and embedded in plastic. Specimens were sectioned, stained and photographed by using a Zeiss EM10C transmission electron microscope.

### Antibodies and reagents

Mouse monoclonal antiserum against LC3 (NanoTools, Teningen, Germany) was used for Western blotting at a dilution of 1∶400; mouse monoclonal antibody against HSV ICP27 (Virusys, Taneytown, MD) was used for indirect immunofluorescence microscopy at a dilution of 1∶1,000; mouse monoclonal antibody against actin (Sigma, St. Louis, MO) was used for Western blotting at a dilution of 1∶2,000; rabbit monoclonal antibody against human phospho-eIF2α (Epitomics, Burlingame, CA) was used for Western blotting at a dilution of 1∶1,000; rabbit polyclonal antiserum against human eIF2α (Santa Cruz Biotechnology, Santa Crux, CA) was used for Western blotting at a dilution of 1∶500; Alexa Fluor 568 conjugated donkey anti-mouse (Molecular Probes, Eugene, OR) was used for indirect immunofluorescence at a dilution of 1∶500; horseradish peroxidase-conjugated goat anti mouse and horseradish peroxidase-conjugated goat anti rabbit (Sigma, St. Louis, MO) were used for Western blotting at a dilution of 1∶10,000. Bafilomycin A, rapamycin and 3-methyladenine were purchased from Sigma, St. Louis, MO.

### Western blots

Cells were washed in cold phosphate-buffered saline (PBS) at 4°C and lysed in a solution containing 10 mM Tris HCl (pH 8.0), 150 mM NaCl, 2 mM EDTA, 1% NP-40, 0.5% sodium deoxycholate and protease inhibitors (Roche, Laval, QC). Nuclei were removed by centrifugation at 13,000 RPM for 10 min at 4°C. Cell lysates were separated by SDS-PAGE and proteins were then transferred to a polyvinylidene fluoride (PVDF) membrane (Millipore, Billerica, MA) that was subsequently blocked with Tris-buffered saline (TBS) containing 0.05% Tween 20 and 1% casein. After blocking, the membranes were probed with appropriate dilutions of primary antibody followed by appropriate dilutions of horseradish peroxidase-conjugated secondary antibody, and proteins were detected using Pierce ECL Western blotting substrate (Thermo Scientific, Rockford, IL) and exposed to film.

### Indirect immunofluorescence

Cells were fixed with 3% paraformaldehyde for 10 min and then permeabilized for 10 minutes with PBS supplemented with 1% bovine serum albumin (PBS/BSA) containing 0.1% Triton X-100. After washing with PBS/BSA, the coverslips were incubated with appropriate dilutions of primary antibody. After subsequent washing with PBS/BSA, the appropriate Alexa Fluor secondary antibody was applied. Nuclei were stained with Hoechst 33342 (Sigma, St. Louis, MO). Images were captured using a Olympus FV1000 laser scanning confocal microscope and Fluoview 1.7.3.0 software through a 60×, 1.42NA, oil immersion objective and a digital zoom factor of 3. Composites are representative images that were assembled using Adobe Photoshop.

### Infectious center assay

L and gro29 cells were grown in 6-well plates with or without glass coverslips before being infected with HSV-1 at an MOI of 10. After 1 h at 37°C, residual virus was inactivated with a low-pH wash (40 mM citric acid [pH 3.0], 10 mM KCl, 135 mM NaCl). At 3 h post infection, cells were rinsed with PBS, trypsinized, and counted. Cell dilutions were then plated immediately onto monolayers of Vero cells in the presence of 0.5% pooled human immunoglobulin. Viral plaques were visualized by staining with 0.5% methylene blue/70% methanol after 3 days of incubation at 37°C. In parallel, gro29 and L cells were seeded on coverslips and were infected as above. At 3 h post infection, cells were fixed and stained, as above, for the immediate early protein ICP27 to determine the proportion of infected cells.

### Live-cell imaging

L and gro29 cells were cultured in glass-bottomed dishes (MatTek Corporation, Ashland, MA) and infected with HSV-1. At 24 h post infection, the medium was replaced with DMEM, without phenol red, containing 10% FBS and the samples were placed in a humidified 37°C chamber in a 5% CO_2_ environment. Images were captured every 3.5 minutes using an Olympus FV1000 laser scanning confocal microscope and Fluoview 1.7.3.0 software through a 40× objective.

## References

[1] Ben-Porat T, Kaplan AS (1985) Molecular biology of pseudorabies virus. In: B.Roizman, editor. The herpesviruses. New York: Plenum Press. pp. 105–173.

[2] Roizman B, Knipe DM (2001) Herpes Simplex Viruses and Their Replication. In: D.M. Knipe PMH, editor. Fields Virology. 4th ed. Philadelphia, Pa: Lippincott Williams & Wilkins. pp. 2399–2459.

[3] Honess RW, Roizman B (1973) Proteins specified by herpes simplex virus. XI. Identification and relative molar rates of synthesis of structural and nonstructural herpes virus polypeptides in the infected cell. J Virol 12: 1347–1365. 4357511 10.1128/jvi.12.6.1347-1365.1973 PMC356776

[4] Mettenleiter TC (2006) Intriguing interplay between viral proteins during herpesvirus assembly or: the herpesvirus assembly puzzle. Vet Microbiol 113: 163–169. 16330166 10.1016/j.vetmic.2005.11.040

[5] Jones F, Grose C (1988) Role of cytoplasmic vacuoles in varicella-zoster virus glycoprotein trafficking and virion envelopment. J Virol 62: 2701–2711. 2839696 10.1128/jvi.62.8.2701-2711.1988 PMC253703

[6] Turcotte S, Letellier J, Lippe R (2005) Herpes simplex virus type 1 capsids transit by the trans-Golgi network, where viral glycoproteins accumulate independently of capsid egress. J Virol 79: 8847–8860. 15994778 10.1128/JVI.79.14.8847-8860.2005 PMC1168770

[7] Whealy ME, Card JP, Meade RP, Robbins AK, Enquist LW (1991) Effect of brefeldin A on alphaherpesvirus membrane protein glycosylation and virus egress. J Virol 65: 1066–1081. 1847436 10.1128/jvi.65.3.1066-1081.1991 PMC239872

[8] Tufaro F, Snider MD, McKnight SL (1987) Identification and characterization of a mouse cell mutant defective in the intracellular transport of glycoproteins. J Cell Biol 105: 647–657. 3040769 10.1083/jcb.105.2.647 PMC2114779

[9] Banfield BW, Tufaro F (1990) Herpes simplex virus particles are unable to traverse the secretory pathway in the mouse L-cell mutant gro29. J Virol 64: 5716–5729. 2173764 10.1128/jvi.64.12.5716-5729.1990 PMC248713

[10] Gruenheid S, Gatzke L, Meadows H, Tufaro F (1993) Herpes simplex virus infection and propagation in a mouse L cell mutant lacking heparan sulfate proteoglycans. J Virol 67: 93–100. 8380101 10.1128/jvi.67.1.93-100.1993 PMC237341

[11] Altenburg JD, Jin Q, Alkhatib B, Alkhatib G (2010) The potent anti-HIV activity of CXCL12gamma correlates with efficient CXCR4 binding and internalization. J Virol 84: 2563–2572. 20015992 10.1128/JVI.00342-09 PMC2820898

[12] Banfield BW, Leduc Y, Esford L, Schubert K, Tufaro F (1995) Sequential isolation of proteoglycan synthesis mutants by using herpes simplex virus as a selective agent: evidence for a proteoglycan-independent virus entry pathway. J Virol 69: 3290–3298. 7745676 10.1128/jvi.69.6.3290-3298.1995 PMC189040

[13] Banfield BW, Leduc Y, Esford L, Visalli RJ, Brandt CR, et al (1995) Evidence for an interaction of herpes simplex virus with chondroitin sulfate proteoglycans during infection. Virology 208: 531–539. 7747425 10.1006/viro.1995.1184

[14] Bender FC, Whitbeck JC, Lou H, Cohen GH, Eisenberg RJ (2005) Herpes simplex virus glycoprotein B binds to cell surfaces independently of heparan sulfate and blocks virus entry. J Virol 79: 11588–11597. 16140736 10.1128/JVI.79.18.11588-11597.2005 PMC1212636

[15] Bengali Z, Townsley AC, Moss B (2009) Vaccinia virus strain differences in cell attachment and entry. Virology 389: 132–140. 19428041 10.1016/j.virol.2009.04.012 PMC2700833

[16] Bergefall K, Trybala E, Johansson M, Uyama T, Naito S, et al (2005) Chondroitin sulfate characterized by the E-disaccharide unit is a potent inhibitor of herpes simplex virus infectivity and provides the virus binding sites on gro2C cells. J Biol Chem 280: 32193–32199. 16027159 10.1074/jbc.M503645200

[17] Chiu WL, Lin CL, Yang MH, Tzou DL, Chang W (2007) Vaccinia virus 4c (A26L) protein on intracellular mature virus binds to the extracellular cellular matrix laminin. J Virol 81: 2149–2157. 17166913 10.1128/JVI.02302-06 PMC1865921

[18] Dyer AP, Banfield BW, Martindale D, Spannier DM, Tufaro F (1997) Dextran sulfate can act as an artificial receptor to mediate a type-specific herpes simplex virus infection via glycoprotein B. J Virol 71: 191–198. 8985338 10.1128/jvi.71.1.191-198.1997 PMC191039

[19] Hung JJ, Hsieh MT, Young MJ, Kao CL, King CC, et al (2004) An external loop region of domain III of dengue virus type 2 envelope protein is involved in serotype-specific binding to mosquito but not mammalian cells. J Virol 78: 378–388. 14671119 10.1128/JVI.78.1.378-388.2004 PMC303388

[20] Jacquet A, Haumont M, Chellun D, Massaer M, Tufaro F, et al (1998) The varicella zoster virus glycoprotein B (gB) plays a role in virus binding to cell surface heparan sulfate proteoglycans. Virus Res 53: 197–207. 9620211 10.1016/s0168-1702(97)00149-4

[21] Laquerre S, Argnani R, Anderson DB, Zucchini S, Manservigi R, et al (1998) Heparan sulfate proteoglycan binding by herpes simplex virus type 1 glycoproteins B and C, which differ in their contributions to virus attachment, penetration, and cell-to-cell spread. J Virol 72: 6119–6130. 9621076 10.1128/jvi.72.7.6119-6130.1998 PMC110418

[22] Mardberg K, Trybala E, Tufaro F, Bergstrom T (2002) Herpes simplex virus type 1 glycoprotein C is necessary for efficient infection of chondroitin sulfate-expressing gro2C cells. J Gen Virol 83: 291–300. 11807221 10.1099/0022-1317-83-2-291

[23] Tal-Singer R, Peng C, Ponce De Leon M, Abrams WR, Banfield BW, et al (1995) Interaction of herpes simplex virus glycoprotein gC with mammalian cell surface molecules. J Virol 69: 4471–4483. 7769707 10.1128/jvi.69.7.4471-4483.1995 PMC189189

[24] Uyama T, Ishida M, Izumikawa T, Trybala E, Tufaro F, et al (2006) Chondroitin 4-O-sulfotransferase-1 regulates E disaccharide expression of chondroitin sulfate required for herpes simplex virus infectivity. J Biol Chem 281: 38668–38674. 17040900 10.1074/jbc.M609320200

[25] Karger A, Saalmuller A, Tufaro F, Banfield BW, Mettenleiter TC (1995) Cell surface proteoglycans are not essential for infection by pseudorabies virus. J Virol 69: 3482–3489. 7745695 10.1128/jvi.69.6.3482-3489.1995 PMC189061

[26] Whealy ME, Robbins AK, Tufaro F, Enquist LW (1992) A cellular function is required for pseudorabies virus envelope glycoprotein processing and virus egress. J Virol 66: 3803–3810. 1316483 10.1128/jvi.66.6.3803-3810.1992 PMC241166

[27] Michaelis C, Banfield BW, Gruenheid S, Tsang Y, Lippe R, et al (1992) Toxin resistance and reduced secretion in a mouse L-cell mutant defective in herpes virus propagation. Biochem Cell Biol 70: 1209–1217. 1338412 10.1139/o92-167

[28] Johnson DC, Spear PG (1982) Monensin inhibits the processing of herpes simplex virus glycoproteins, their transport to the cell surface, and the egress of virions from infected cells. J Virol 43: 1102–1112. 6292453 10.1128/jvi.43.3.1102-1112.1982 PMC256222

[29] Klionsky DJ, Emr SD (2000) Autophagy as a regulated pathway of cellular degradation. Science 290: 1717–1721. 11099404 10.1126/science.290.5497.1717 PMC2732363

[30] Dever TE, Feng L, Wek RC, Cigan AM, Donahue TF, et al (1992) Phosphorylation of initiation factor 2 alpha by protein kinase GCN2 mediates gene-specific translational control of GCN4 in yeast. Cell 68: 585–596. 1739968 10.1016/0092-8674(92)90193-g

[31] Talloczy Z, Jiang W, Virgin HWt, Leib DA, Scheuner D, et al (2002) Regulation of starvation- and virus-induced autophagy by the eIF2alpha kinase signaling pathway. Proc Natl Acad Sci U S A 99: 190–195. 11756670 10.1073/pnas.012485299 PMC117537

[32] Deretic V (2011) Autophagy in immunity and cell-autonomous defense against intracellular microbes. Immunol Rev 240: 92–104. 21349088 10.1111/j.1600-065X.2010.00995.x PMC3057454

[33] Deretic V, Levine B (2009) Autophagy, immunity, and microbial adaptations. Cell Host Microbe 5: 527–549. 19527881 10.1016/j.chom.2009.05.016 PMC2720763

[34] Virgin HW, Levine B (2009) Autophagy genes in immunity. Nat Immunol 10: 461–470. 19381141 10.1038/ni.1726 PMC2715365

[35] Cassady KA, Gross M (2002) The herpes simplex virus type 1 U(S)11 protein interacts with protein kinase R in infected cells and requires a 30-amino-acid sequence adjacent to a kinase substrate domain. J Virol 76: 2029–2035. 11836380 10.1128/jvi.76.5.2029-2035.2002 PMC135940

[36] Chaumorcel M, Souquere S, Pierron G, Codogno P, Esclatine A (2008) Human cytomegalovirus controls a new autophagy-dependent cellular antiviral defense mechanism. Autophagy 4: 46–53. 18340111 10.4161/auto.5184

[37] Gannage M, Dormann D, Albrecht R, Dengjel J, Torossi T, et al (2009) Matrix protein 2 of influenza A virus blocks autophagosome fusion with lysosomes. Cell Host Microbe 6: 367–380. 19837376 10.1016/j.chom.2009.09.005 PMC2774833

[38] Jackson WT, Giddings TH Jr, Taylor MP, Mulinyawe S, Rabinovitch M, et al (2005) Subversion of cellular autophagosomal machinery by RNA viruses. PLoS Biol 3: e156. 15884975 10.1371/journal.pbio.0030156 PMC1084330

[39] Kyei GB, Dinkins C, Davis AS, Roberts E, Singh SB, et al (2009) Autophagy pathway intersects with HIV-1 biosynthesis and regulates viral yields in macrophages. J Cell Biol 186: 255–268. 19635843 10.1083/jcb.200903070 PMC2717652

[40] Orvedahl A, Alexander D, Talloczy Z, Sun Q, Wei Y, et al (2007) HSV-1 ICP34.5 confers neurovirulence by targeting the Beclin 1 autophagy protein. Cell Host Microbe 1: 23–35. 18005679 10.1016/j.chom.2006.12.001

[41] Sinha S, Colbert CL, Becker N, Wei Y, Levine B (2008) Molecular basis of the regulation of Beclin 1-dependent autophagy by the gamma-herpesvirus 68 Bcl-2 homolog M11. Autophagy 4: 989–997. 18797192 10.4161/auto.6803 PMC2755179

[42] Taylor MP, Kirkegaard K (2007) Modification of cellular autophagy protein LC3 by poliovirus. J Virol 81: 12543–12553. 17804493 10.1128/JVI.00755-07 PMC2169029

[43] Chou J, Roizman B (1992) The gamma 1(34.5) gene of herpes simplex virus 1 precludes neuroblastoma cells from triggering total shutoff of protein synthesis characteristic of programed cell death in neuronal cells. Proc Natl Acad Sci U S A 89: 3266–3270. 1314384 10.1073/pnas.89.8.3266 PMC48847

[44] He B, Gross M, Roizman B (1997) The gamma(1)34.5 protein of herpes simplex virus 1 complexes with protein phosphatase 1alpha to dephosphorylate the alpha subunit of the eukaryotic translation initiation factor 2 and preclude the shutoff of protein synthesis by double-stranded RNA-activated protein kinase. Proc Natl Acad Sci U S A 94: 843–848. 9023344 10.1073/pnas.94.3.843 PMC19601

[45] He B, Gross M, Roizman B (1998) The gamma134.5 protein of herpes simplex virus 1 has the structural and functional attributes of a protein phosphatase 1 regulatory subunit and is present in a high molecular weight complex with the enzyme in infected cells. J Biol Chem 273: 20737–20743. 9694816 10.1074/jbc.273.33.20737

[46] Mizushima N, Yoshimori T, Levine B (2010) Methods in mammalian autophagy research. Cell 140: 313–326. 20144757 10.1016/j.cell.2010.01.028 PMC2852113

[47] Kabeya Y, Mizushima N, Ueno T, Yamamoto A, Kirisako T, et al (2000) LC3, a mammalian homologue of yeast Apg8p, is localized in autophagosome membranes after processing. EMBO J 19: 5720–5728. 11060023 10.1093/emboj/19.21.5720 PMC305793

[48] Kabeya Y, Mizushima N, Yamamoto A, Oshitani-Okamoto S, Ohsumi Y, et al (2004) LC3, GABARAP and GATE16 localize to autophagosomal membrane depending on form-II formation. J Cell Sci 117: 2805–2812. 15169837 10.1242/jcs.01131

[49] Tanida I, Minematsu-Ikeguchi N, Ueno T, Kominami E (2005) Lysosomal turnover, but not a cellular level, of endogenous LC3 is a marker for autophagy. Autophagy 1: 84–91. 16874052 10.4161/auto.1.2.1697

[50] Mizushima N, Yamamoto A, Matsui M, Yoshimori T, Ohsumi Y (2004) In vivo analysis of autophagy in response to nutrient starvation using transgenic mice expressing a fluorescent autophagosome marker. Mol Biol Cell 15: 1101–1111. 14699058 10.1091/mbc.E03-09-0704 PMC363084

[51] Kuma A, Matsui M, Mizushima N (2007) LC3, an autophagosome marker, can be incorporated into protein aggregates independent of autophagy: caution in the interpretation of LC3 localization. Autophagy 3: 323–328. 17387262 10.4161/auto.4012

[52] Mizushima N, Yoshimori T (2007) How to interpret LC3 immunoblotting. Autophagy 3: 542–545. 17611390 10.4161/auto.4600

[53] Yamamoto A, Tagawa Y, Yoshimori T, Moriyama Y, Masaki R, et al (1998) Bafilomycin A1 prevents maturation of autophagic vacuoles by inhibiting fusion between autophagosomes and lysosomes in rat hepatoma cell line, H-4-II-E cells. Cell Struct Funct 23: 33–42. 9639028 10.1247/csf.23.33

[54] Wullschleger S, Loewith R, Hall MN (2006) TOR signaling in growth and metabolism. Cell 124: 471–484. 16469695 10.1016/j.cell.2006.01.016

[55] Seglen PO, Gordon PB (1982) 3-Methyladenine: specific inhibitor of autophagic/lysosomal protein degradation in isolated rat hepatocytes. Proc Natl Acad Sci U S A 79: 1889–1892. 6952238 10.1073/pnas.79.6.1889 PMC346086

[56] Suarez AL, Kong R, George T, He L, Yue Z, et al (2011) Gammaherpesvirus 68 infection of endothelial cells requires both host autophagy genes and viral oncogenes for optimal survival and persistence. J Virol 85: 6293–6308. 21490089 10.1128/JVI.00001-11 PMC3126501

[57] Yang Z, Klionsky DJ (2009) An overview of the molecular mechanism of autophagy. Curr Top Microbiol Immunol 335: 1–32. 19802558 10.1007/978-3-642-00302-8_1 PMC2832191

[58] Antinone SE, Zaichick SV, Smith GA (2010) Resolving the assembly state of herpes simplex virus during axon transport by live-cell imaging. J Virol 84: 13019–13030. 20810730 10.1128/JVI.01296-10 PMC3004300

[59] McFarlane S, Aitken J, Sutherland JS, Nicholl MJ, Preston VG, et al (2011) Early induction of autophagy in human fibroblasts after infection with human cytomegalovirus or herpes simplex virus 1. J Virol 85: 4212–4221. 21325419 10.1128/JVI.02435-10 PMC3126239

[60] McCormick C, Duncan G, Goutsos KT, Tufaro F (2000) The putative tumor suppressors EXT1 and EXT2 form a stable complex that accumulates in the Golgi apparatus and catalyzes the synthesis of heparan sulfate. Proc Natl Acad Sci U S A 97: 668–673. 10639137 10.1073/pnas.97.2.668 PMC15388

[61] McCormick C, Leduc Y, Martindale D, Mattison K, Esford LE, et al (1998) The putative tumour suppressor EXT1 alters the expression of cell-surface heparan sulfate. Nat Genet 19: 158–161. 9620772 10.1038/514

[62] Alexander DE, Leib DA (2008) Xenophagy in herpes simplex virus replication and pathogenesis. Autophagy 4: 101–103. 18000391 10.4161/auto.5222 PMC3775468

[63] Kudchodkar SB, Levine B (2009) Viruses and autophagy. Rev Med Virol 19: 359–378. 19750559 10.1002/rmv.630 PMC2852112

[64] Sandvig K, van Deurs B (1992) Toxin-induced cell lysis: protection by 3-methyladenine and cycloheximide. Exp Cell Res 200: 253–262. 1572394 10.1016/0014-4827(92)90171-4

[65] Lippe R, Kolaitis G, Michaelis C, Tufaro F, Jefferies WA (1993) Differential recruitment of viral and allo-epitopes into the MHC class I antigen processing pathway of a novel mutant of Ltk- cells. HSV/MHC class I restriction/immune recognition/antigen processing/antigen presentation/influenza virus. J Immunol 150: 3170–3179. 7682234

[66] Zhang QJ, Chen SS, Saari CA, Massuci MG, Tufaro F, et al (2000) Evidence of selective processing of immunodominant epitopes in virally infected cells. J Immunol 164: 4513–4521. 10779752 10.4049/jimmunol.164.9.4513

[67] Guo JY, Chen HY, Mathew R, Fan J, Strohecker AM, et al (2011) Activated Ras requires autophagy to maintain oxidative metabolism and tumorigenesis. Genes Dev 25: 460–470. 21317241 10.1101/gad.2016311 PMC3049287

[68] Kim MJ, Woo SJ, Yoon CH, Lee JS, An S, et al (2011) Involvement of autophagy in oncogenic K-Ras-induced malignant cell transformation. J Biol Chem 286: 12924–12932. 21300795 10.1074/jbc.M110.138958 PMC3075639

[69] Lock R, Roy S, Kenific CM, Su JS, Salas E, et al (2011) Autophagy facilitates glycolysis during Ras-mediated oncogenic transformation. Mol Biol Cell 22: 165–178. 21119005 10.1091/mbc.E10-06-0500 PMC3020913

[70] Yang S, Wang X, Contino G, Liesa M, Sahin E, et al (2011) Pancreatic cancers require autophagy for tumor growth. Genes Dev 25: 717–729. 21406549 10.1101/gad.2016111 PMC3070934

[71] Garcin D, Michal Y, Jault F, Lyon M, Lenoir G, et al (1990) Inhibition of HSV-1 multiplication in rat embryo fibroblasts constitutively expressing the EJ-ras oncogene. Virology 179: 208–216. 2171205 10.1016/0042-6822(90)90290-8

[72] Rusten TE, Stenmark H (2009) How do ESCRT proteins control autophagy? J Cell Sci 122: 2179–2183. 19535733 10.1242/jcs.050021

[73] Calistri A, Sette P, Salata C, Cancellotti E, Forghieri C, et al (2007) Intracellular trafficking and maturation of herpes simplex virus type 1 gB and virus egress require functional biogenesis of multivesicular bodies. J Virol 81: 11468–11478. 17686835 10.1128/JVI.01364-07 PMC2045546

[74] Crump CM, Yates C, Minson T (2007) Herpes simplex virus type 1 cytoplasmic envelopment requires functional Vps4. J Virol 81: 7380–7387. 17507493 10.1128/JVI.00222-07 PMC1933334

[75] Pawliczek T, Crump CM (2009) Herpes simplex virus type 1 production requires a functional ESCRT-III complex but is independent of TSG101 and ALIX expression. J Virol 83: 11254–11264. 19692479 10.1128/JVI.00574-09 PMC2772754

[76] Swift S, Lorens J, Achacoso P, Nolan GP (2001) Rapid production of retroviruses for efficient gene delivery to mammalian cells using 293T cell-based systems. Curr Protoc Immunol Chapter 10: Unit 10 17C. 10.1002/0471142735.im1017cs31 18432682

[77] Tanaka M, Kagawa H, Yamanashi Y, Sata T, Kawaguchi Y (2003) Construction of an excisable bacterial artificial chromosome containing a full-length infectious clone of herpes simplex virus type 1: viruses reconstituted from the clone exhibit wild-type properties in vitro and in vivo. J Virol 77: 1382–1391. 12502854 10.1128/JVI.77.2.1382-1391.2003 PMC140785

[78] Tischer BK, Smith GA, Osterrieder N (2010) En passant mutagenesis: a two step markerless red recombination system. Methods Mol Biol 634: 421–430. 20677001 10.1007/978-1-60761-652-8_30

[79] Finnen RL, Roy BB, Zhang H, Banfield BW (2010) Analysis of filamentous process induction and nuclear localization properties of the HSV-2 serine/threonine kinase Us3. Virology 397: 23–33. 19945726 10.1016/j.virol.2009.11.012 PMC2813931

